# Changes in body water distribution and phase angle following rapid ascent to 3,680 m and their association with acute mountain sickness: a prospective cohort study

**DOI:** 10.3389/fpubh.2025.1742613

**Published:** 2026-01-14

**Authors:** Xianglian Li, Jie He, Jie Zhang, Yaru Li, Yanlin Zhu, Liwen Mo, Yue Cheng

**Affiliations:** 1 Department of Nephrology, General Hospital of Western Theater Command of PLA, Chengdu, China; 2 College of Medicine, Southwest Jiaotong University, Chengdu, China

**Keywords:** acute mountain sickness, bioelectrical impedance analysis, body composition, body water, ECW/ICW, phase angle

## Abstract

**Background:**

Acute mountain sickness (AMS) is a common pathological response following rapid ascent to high altitude, and its underlying mechanisms remain incompletely understood. This study focused on investigating the early changes in body composition following acute high-altitude exposure and their association with AMS.

**Methods:**

A prospective cohort study was conducted on healthy individuals who flew from plain (500 m) to plateau (3,680 m). Multi-frequency bioelectrical impedance analysis was used to measure body composition indicators within 24 h before and 24 h after ascent. AMS was diagnosed and its severity assessed using the Lake Louise Score System (LLSS). Comparisons of body composition changes before and after high-altitude exposure were performed, and intergroup comparisons were conducted based on the occurrence of AMS. Spearman correlation analysis and multiple linear regression were used to explore the relationships between body composition changes and LLSS score.

**Results:**

A total of 34 participants were included, and the AMS incidence was 61.76%. Compared with the plain baseline, after rapid ascent to high altitude, extracellular-to-intracellular water ratio (ECW/ICW) (*t* = −2.41, *p* = 0.022) and phase angle (PhA) (*t* = −4.78, *p* < 0.001) decreased significantly, and participants with AMS exhibited greater magnitude decrease in both ΔECW/ICW (*Z* = −2.14, *p* = 0.032) and ΔPhA (*Z* = −3.01, *p* = 0.003). Spearman correlation analysis showed that both ΔECW/ICW (*r* = −0.447, *p* = 0.008) and ΔPhA (*r* = −0.646, *p* < 0.001) were negatively correlated with the LLSS score. However, all results of ECW/ICW and ΔECW/ICW shown no statistically significant after Bonferroni correction. Multiple linear regression analysis indicated that only ΔPhA was an independent predictor of the LLSS score (*β* = −0.507, *p* = 0.001).

**Conclusion:**

After rapid exposure to high altitude, body water often shifted into cells and pronounced cellular-level dysfunction occurred. Susceptible individuals with AMS exhibited a more pronounced decrease in PhA. The change of PhA (ΔPhA), rather than the change of ECW/ICW (ΔECW/ICW), was an independent predictor of AMS severity. Monitoring ΔPhA may provide an effective, non-invasive method for early risk warning of AMS.

## 1 Introduction

Acute mountain sickness (AMS) is the most common clinical syndrome occurring after rapid ascent to a high-altitude hypoxic environment (usually refers to rising from low altitude to ≥2,500 m within 24 h) (1). The typical symptoms include headache, nausea, dizziness, fatigue and sleep disorder, which usually appear several hours after reaching high altitude, and gradually worsen, reaching a peak within 18 to 24 h. Most of symptoms disappear on the second or third day, and a few severe cases may progress to high altitude brain edema or high altitude pulmonary edema, threatening life (2–4). With the increase of plateau tourism and work, the prevention and management of AMS has become an important challenge in the field of public health.

Changes in body water balance have long been seen as one of the key links to AMS occurrence (5). Traditional belief suggests sudden low oxygen can excite the nervous system, activate the renin–angiotensin–aldosterone system (RAAS), increase secretion of antidiuretic hormone, and finally lead to water and sodium retention (6, 7). Meanwhile, the expression of vascular endothelial growth factor induced by hypoxia mediates not only microvascular permeability, but also promotes the transfer of body water from blood vessels to tissue spaces and cells, causing tissue edema, especially brain edema, resulting in a series of symptoms of AMS (8). Nonetheless, AMS’s exact mechanisms remain unclear, and studies on body water state after sudden high altitude exposure are lacking, with mixed outcomes. Some research shows water retention starts 3–12 h after hypoxia exposure, linking it to AMS symptoms (9, 10). Other studies suggested that water retention is not a necessary factor for AMS development (11). However, some researchers reported that dehydration can aggravate AMS symptoms, emphasized the importance of active hydration (12, 13). These varied findings could stem from differences in population characteristics (such as age, physical fitness, acclimatization history), exposure conditions (rising rate, absolute altitude, residence time) and body water assessment methods (such as bioelectrical impedance, body weight change, hormone level).

Bioelectrical impedance analysis (BIA) technology is useful for assessing body composition and body water distribution without any invasive procedures. It works quickly and gives measurable results (14). Compared with traditional methods, multi-frequency BIA can distinguish extracellular water (ECW) from intracellular water (ICW) and derive the index of phase angle (PhA) (15–17). The ratio of ECW to ICW (ECW/ICW) is a key index to evaluate the body water distribution inside and outside cells (18). A lower ratio means more water moving inside cells. PhA can directly reflect the integrity of the cell membrane and the cell functional state. If PhA decreases, it means that cell health is poorer (16, 19). Body composition will change in a hypoxic environment (20–22). Most studies focus on the description of body composition at a static or single time point and do not fully describe the changes in body water distribution and track of cell function when moving quickly to rapid high-altitude ascent. These studies also do not deeply explore how these changes relate to AMS. This lack of detail stops us from fully understanding the early stages of AMS by looking at cell and fluid changes.

Therefore, this prospective cohort study was employed to observe the dynamic changes in body composition during the early phase after rapid ascent to high altitude. The focus was on how water is distributed between inside and outside of cells, as well as changes in cell activities. This was done to see how these factors relate to the start and development of AMS. Multi-frequency BIA was used to monitor the body composition parameters in healthy individuals before and 24 h after rapid ascent to high altitude. We aimed to: (1) Describe the early dynamic changes in body composition such as ECW/ICW and PhA following rapid ascent. (2) Analyze the correlation between these dynamic changes and AMS severity (assessed by the Lake Louise Score). (3) Preliminarily evaluate if these changes can predict AMS. This research is expected to provide new insights by looking at how water distribution and cell function relate to AMS, which has not been clear before. Additionally, it aims to create a simple method to warn of AMS risk using BIA.

## 2 Methods

### 2.1 Study design and participants

A prospective cohort study was conducted (Supplementary Figure 1). Healthy individuals from our hospital who had a short mission at high altitude were enrolled through a special recruitment process from April to June 2024. They would plan to fly from the Chengdu Plain (500 m) to a high-altitude area at 3,680 m in July 2024, the flight would take 2.5 h. Before starting, everyone was told about the study procedures. Researchers met them in person to make sure they understood. After they agreed, they signed a consent form. Then, they all had a health check at our hospital. To join the study, participants had to be 18–50 years old, have a normal health check, and not have gone to high altitude in the past year. People could not join if they had cardiovascular, cerebrovascular, respiratory, digestive, urinary, or endocrine system diseases, chronic headache/migraine, or had used medicine (e.g., corticosteroids, acetazolamide, non-steroidal anti-inflammatory drugs, *Rhodiola rosea*, diuretics) to prevent AMS within 1 week before the study or during ascent. Everyone had to agree in writing to be part of the study. The hospital’s ethical board allowed the study (No. 2024EC-ky001).

### 2.2 Measurements and data collection

Information such as name, age, gender, height, medical past, and medication history was gathered a week before participants went to high altitude. All participants were accommodated in a dedicated research living area during the whole study process. They were centrally managed to ensure the standardization of diet, daily routine, and physical activity. Meals were uniformly provided by the Nutrition Department, offering standardized breakfast, lunch, and dinner. Participants could not eat or drink anything from outside. They were not allowed to smoke, drink alcohol, or have drinks with caffeine or theophylline like coffee, tea, or cola. A guide called “Participant Activity Guideline” was shared with them which stopped them from doing exercise, heavy work, or walking long distances. They had to sleep properly every day with lights out at 22:30 and waking up at 06:30, checked by the area manager. During the whole time from starting measurements on plains to the finishing measurements after high altitude, no one used extra oxygen, hyperbaric oxygen therapy, or portable pressure devices. This made sure that any changes in their bodies were due to high-altitude hypoxic exposure.

A multi-frequency BIA device (InBody S10, Biospace Inc., South Korea) was used. Before starting the study, the device was set up correctly by following the company’s instructions. Tests took place indoors where the temperature was kept between 22 °C and 24 °C, from 6:30 a.m. to 8:30 a.m. Participants stopped eating after 10:00 p.m. but could drink some plain water. Before being measured, participants emptied their bladder and bowels, wore light cotton clothing without metal, took off shoes, and sat for at least 10 min. The same trained person did all the measurements, following strict rules. The measured parameters included body weight (BW), body mass index (BMI), extracellular water (ECW), intracellular water (ICW), total body water (TBW), ECW/ICW, phase angle (PhA), fat mass (FAT), fat-free mass (FFM), protein, mineral, skeletal muscle mass (SMM), percentage body fat (PBF), and basal metabolic rate (BMR). Measurements were taken 24 h before arriving to the high altitude (as the plain baseline) and 24 h after arrival (as a high-altitude measure). The changes in body composition (Δ value) were calculated by subtracting the plain measure from the high-altitude measure.

AMS was assessed using the Lake Louise Scoring System (LLSS) (23). The assessment was performed before any BIA tests. Two researchers with the same training carried out the interview assessment together. If there was a scoring disagreement, a more experienced researcher made the final decision. The BIA measurements and the AMS assessments were conducted by separate researchers, at different time points, and data were recorded on independent case report forms before being merged for analysis. The LLSS checked for symptoms like headache, gastrointestinal symptoms, fatigue/weakness, and dizziness/light-headedness. AMS was defined as a headache score ≥1 and a total score ≥3. The severity was classified as mild (3–5 points), moderate (6–9 points), or severe (≥10 points).

### 2.3 Statistical analysis

Statistical analyses were performed using IBM SPSS Statistics version 25.0. Categorical variables were presented as frequencies and percentages, and group comparisons were made using the χ^2^ test or Fisher’s exact test. Continuous variables were first tested for normality using the Shapiro–Wilk test. Normally distributed data were described as means ± SDs. We compared two different groups with the independent samples *t*-test, and for two related samples, we used the paired samples *t*-test. Non-normally distributed data were described as median (P25, P75). For comparison of two groups with non-normal data, we used the Mann–Whitney U test, and for related samples, we used the Wilcoxon signed-rank test. Spearman correlation analysis was used to explore the associations between body composition parameters and LLSS score. We further used multiple linear regression to see what factors affect the LLSS score. A *p*-value < 0.05 was considered statistically significant. To control for potential false positives resulting from multiple comparisons, Bonferroni correction was applied, with the adjusted significance level at 0.05 divided by the total number of tests.

Finally, multiple linear regression was employed to explore how changes in body composition might predict AMS. The regression analysis needed at least 10 samples per variable. Potential variables with *p* < 0.05 in the Spearman correlation analysis were first screened, and then combined these with known clinical factors into the regression model as covariates.

## 3 Results

### 3.1 Demographic characteristics and AMS incidence

The study tracked a group of 34 individuals, including 22 males and 12 females, aged between 23 and 47 years old, with a mean age of 32.85 ± 5.46 years. After a rapid ascent to high altitude, 21 individuals developed AMS, yielding an incidence rate of 61.8%. Among them, 18 cases (52.9%) had mild sickness, and 3 cases (8.8%) had moderate sickness (Supplementary Tables 1, 2).

### 3.2 Changes in body composition after rapid ascent to high altitude

Before correction for multiple comparisons, when compared to the plain baseline values, individuals showed a significant decrease in ECW/ICW (*t* = −2.41, *p* = 0.022) and PhA (*t* = −4.78, *p* < 0.001), while FFM increased (*t* = 2.47, *p* = 0.019) after a 24 h exposure to high altitude. No statistically significant differences were observed for BW, BMI, ECW, ICW, TBW, FAT, protein, mineral, SMM, PBF, BMR (*p* > 0.05). After using Bonferroni correction, the significance level was set at 0.0036 (*α* = 0.05/14). Results showed that only the decrease in PhA remained statistically significant (*p* < 0.001). Meanwhile, the differences in ECW/ICW and FFM were no longer statistically significant (Table 1).

**Table 1:** Changes in body composition in the overall population before and after rapid ascent to high altitude (*n* = 34).

Variable	Plain area (500 m)	High altitude (3,680 m)	*t*/*Z*	*p*	Bonferroni correction (adjusted *α* = 0.0036)
Weight (kg)	67.30 ± 10.96	67.29 ± 10.96	−0.17^a^	0.869	ns
BMI (kg/m^2^)	23.07 ± 2.57	23.07 ± 2.58	−0.16^a^	0.871	ns
ECW (L)	15.41 ± 2.97	15.30 ± 2.87	−1.24^a^	0.224	ns
ICW (L)	25.58 ± 5.33	25.59 ± 5.19	0.08^a^	0.936	ns
TBW (L)	40.98 ± 8.28	40.88 ± 8.03	-0.55^a^	0.586	ns
ECW/ICW	0.61 ± 0.02	0.60 ± 0.02	−2.41^a^	0.022^*^	ns
FAT (kg)	11.65 (7.70, 14.10)	11.80 (7.20, 13.80)	−0.36^b^	0.721	ns
FFM (kg)	55.63 ± 11.05	56.05 ± 11.04	2.47^a^	0.019^*^	ns
Protein (kg)	11.07 ± 2.31	11.05 ± 2.24	−0.41^a^	0.685	ns
Mineral (kg)	4.09 ± 0.82	4.11 ± 0.77	−0.26^a^	0.794	ns
SMM (kg)	31.36 ± 6.96	31.35 ± 6.76	−0.08^a^	0.936	ns
PBF (%)	17.36 ± 6.63	17.23 ± 6.75	−0.40^a^	0.695	ns
BMR (kcal)	1,582.88 ± 246.07	1,580.53 ± 238.68	−0.44^a^	0.662	ns
PhA (°)	6.36 ± 0.88	6.24 ± 0.84	−4.78^a^	<0.001^*^	*p* < 0.0036^**^

a Paired-samples *t* test.

b Wilcoxon signed-rank test.

*p < 0.05; **significant after Bonferroni correction; ns, not significant after Bonferroni correction.

### 3.3 The differences in body composition between the AMS+ and AMS− group

There were no significant differences were observed between the AMS+ and AMS− groups in terms of age, gender, and baseline body composition on the plain. Although unadjusted analysis indicated a trend toward lower ECW/ICW ratios in the AMS+ group after rapid high-altitude exposure (*t* = −2.14, *p* = 0.040), this difference was no longer statistically significant following Bonferroni correction. This indicates that, after controlling for the risk of false positives, no significant differences in body composition were observed between AMS+ and AMS− individuals in this study, either at plain or after exposure to high altitude (Table 2).

**Table 2:** Comparison of body composition between AMS+ and AMS− groups.

Variable	Plain area (500 m)					High altitude (3,680 m)				
	AMS+ (*n* = 21)	AMS− (*n* = 13)	*t/χ^2^*	*p*	Bonferroni correction (adjusted *α* = 0.0031)	AMS+ (*n* = 21)	AMS− (*n* = 13)	*t*	*p*	Bonferroni correction (adjusted *α* = 0.0036)
Age (year)	32.19 ± 4.76	33.92 ± 6.49	−0.90^a^	0.376	ns	–	–	–	–	–
Gender (male)	13 (61.9%)	9 (69.2%)	–^b^	0.727	ns	–	–	–	–	–
BW (kg)	66.39 ± 11.72	68.77 ± 9.79	−0.61^a^	0.546	ns	66.44 ± 11.57	68.65 ± 10.18	−0.57^a^	0.575	ns
BMI (kg/m^2^)	23.07 ± 2.88	23.09 ± 2.10	−0.02^a^	0.982	ns	23.09 ± 2.81	23.04 ± 2.24	0.05^a^	0.962	ns
ECW (L)	15.23 ± 3.19	15.68 ± 2.69	−0.43^a^	0.674	ns	15.06 ± 2.97	15.68 ± 2.97	−0.60^a^	0.551	ns
ICW (L)	25.41 ± 5.84	25.85 ± 4.60	−0.23^a^	0.820	ns	25.49 ± 5.62	25.75 ± 4.62	−0.14^a^	0.889	ns
TBW (L)	40.64 ± 9.01	41.53 ± 7.26	−0.30^a^	0.766	ns	40.55 ± 8.58	41.42 ± 7.38	−0.30^a^	0.763	ns
ECW/ICW	0.60 ± 0.02	0.61 ± 0.02	−0.78^a^	0.439	ns	0.59 ± 0.02	0.61 ± 0.01	−2.14^a^	0.040^*^	ns
FAT (kg)	11.48 ± 5.54	12.09 ± 3.18	−0.36^a^	0.720	ns	11.49 ± 6.06	11.91 ± 3.21	−0.23^a^	0.821	ns
FFM (kg)	55.01 ± 11.95	56.63 ± 9.79	−0.41^a^	0.685	ns	55.64 ± 11.80	56.71 ± 10.11	−0.27^a^	0.788	ns
Protein (kg)	11.00 ± 2.53	11.18 ± 2.00	−0.22^a^	0.825	ns	10.10 ± 2.43	11.14 ± 1.99	−0.18^a^	0.860	ns
Mineral (kg)	4.08 ± 0.91	4.12 ± 0.67	−0.12^a^	0.907	ns	4.09 ± 0.80	4.15 ± 0.76	−0.21^a^	0.835	ns
SMM (kg)	31.15 ± 7.62	31.72 ± 6.01	−0.23^a^	0.821	ns	31.23 ± 7.31	31.55 ± 6.04	−0.13^a^	0.894	ns
PBF (%)	17.11 ± 7.49	17.76 ± 5.21	−0.27^a^	0.787	ns	17.01 ± 7.79	17.58 ± 4.87	−0.24^a^	0.816	ns
BMR (kcal)	1,573.52 ± 268.69	1,598.00 ± 213.98	−0.28^a^	0.783	ns	1,571.71 ± 255.24	1,594.77 ± 218.48	−0.27^a^	0.789	ns
PhA (°)	6.47 ± 1.03	6.18 ± 0.56	0.92^a^	0.365	ns	6.30 ± 0.99	6.15 ± 0.54	0.47^a^	0.640	ns

a 2-sample *t* test.

b Fisher exact test.

*p < 0.05; ns, not significant after Bonferroni correction.

### 3.4 Comparison of the magnitude of body composition changes (Δ value) before and after rapid ascent to high altitude between the AMS+ and AMS− groups

The AMS+ group showed a significantly greater decrease in both ECW/ICW (*Z* = −2.141, *p* = 0.032) and PhA (*Z* = −3.012, *p* = 0.003) compared to the AMS− group. No significant differences were detected in the magnitude changes of the other body composition (*p* > 0.05). After using the Bonferroni correction, only ΔPhA remained statistically significant (Table 3). The AMS+ group showed a more pronounced cellular-level dysfunction, suggesting that ΔPhA, as an indicator of cellular integrity, demonstrates superior discriminatory power for AMS compared to ΔECW/ICW.

**Table 3:** Comparison of the magnitude of change (Δ) in body composition after rapid ascent to high altitude between the AMS+ and AMS− group.

Variable	AMS+ (*n* = 21)	AMS− (*n* = 13)	*t*/*Z*	*p*	Bonferroni correction (adjusted *α* = 0.0036)
ΔBW (kg)	0.00 (0.00, 0.00)	0.00 (0.00, 0.00)	−0.85^a^	0.398	ns
ΔBMI (kg/m^2^)	0.00 (0.00, 0.00)	0.00 (0.00, 0.00)	−0.94^a^	0.349	ns
ΔECW (L)	−0.17 ± 0.41	−0.01 ± 0.65	−0.90^b^	0.373	ns
ΔICW (L)	0.10 (−0.20, 0.60)	0.00 (−0.50, 0.20)	−0.94^a^	0.347	ns
ΔTBW (L)	−0.10 (−0.40, 0.50)	−0.10 (−0.70, 0.20)	−0.32^a^	0.749	ns
Δ ECW/ICW	−0.01 (−0.01, 0.00)	0.00 (−0.01, 0.01)	−2.14^a^	0.032^*^	ns
ΔFAT (kg)	0.01 ± 1.22	−0.18 ± 1.78	0.38^b^	0.707	ns
ΔFFM (kg)	0.60 ± 0.82	0.22 ± 1.14	1.11^b^	0.277	ns
ΔProtein (kg)	0.00 (−0.10, 0.30)	0.00 (−0.20, 0.10)	−0.50^a^	0.615	ns
ΔMineral (kg)	0.06 (−0.02, 0.10)	0.03 (−0.01, 0.10)	−0.02^a^	0.986	ns
ΔSMM (kg)	0.10 (−0.20, 0.80)	0.00 (−0.70, 0.20)	−1.01^a^	0.311	ns
ΔPBF (%)	−0.10 ± 1.78	−0.18 ± 2.38	0.11^b^	0.912	ns
ΔBMR (kcal)	1.00 (−10.00, 19.00)	−6.00 (−23.00, 4.00)	−0.25^a^	0.804	ns
ΔPhA (°)	−0.20 (−0.30, −0.10)	−0.10 (−0.10, 0.00)	−3.01^a^	0.003^*^	*p* < 0.0036^**^

a Mann–Whitney U test.

b 2-sample *t* test.

*p < 0.05; ns, not significant after Bonferroni correction; **significant after Bonferroni correction.

### 3.5 Correlation between body composition and AMS symptom scores

Spearman analysis showed that body composition neither at the plain nor measured after rapid ascent to high altitude was correlated with the LLSS score. However, ΔECW/ICW showed a negative correlation with the LLSS score (*r* = −0.447, *p* = 0.008), and ΔPhA also demonstrated a significant negative correlation with the LLSS score (*r* = −0.646, *p* < 0.001). But, after applying Bonferroni correction, only ΔPhA kept a strong negative link with the LLSS score (Table 4).

**Table 4:** Spearman correlation analysis between body composition and LLSS scores.

Variable	Plain area (500 m)			High altitude (3,680 m)			Δ value		
	*r*	*p*	Bonferroni correction (adjusted *α* = 0.0031)	*r*	*p*	Bonferroni correction (adjusted *α* = 0.0036)	*r*	*p*	Bonferroni correction (adjusted *α* = 0.0036)
Age (year)	−0.326	0.06	ns	–	–	–	–	–	–
Gender	−0.113	0.525	ns	–	–	–	–	–	–
BW (kg)	−0.313	0.072	ns	−0.301	0.084	ns	0.218	0.215	ns
BMI (kg/m^2^)	−0.226	0.199	ns	−0.196	0.266	ns	0.229	0.192	ns
ECW (L)	−0.229	0.192	ns	−0.226	0.199	ns	−0.066	0.712	ns
ICW (L)	−0.216	0.22	ns	−0.171	0.333	ns	0.233	0.184	ns
TBW (L)	−0.221	0.208	ns	−0.194	0.272	ns	0.122	0.493	ns
ECW/ICW	0.043	0.808	ns	−0.288	0.099	ns	−0.447	0.008*	ns
FAT (kg)	−0.331	0.056	ns	−0.308	0.077	ns	0.030	0.867	ns
FFM (kg)	−0.223	0.206	ns	−0.194	0.272	ns	0.160	0.365	ns
Protein (kg)	−0.210	0.233	ns	−0.182	0.304	ns	0.162	0.360	ns
Mineral (kg)	−0.199	0.258	ns	−0.185	0.295	ns	−0.034	0.850	ns
SMM (kg)	−0.215	0.223	ns	−0.174	0.326	ns	0.241	0.169	ns
PBF (%)	−0.246	0.161	ns	−0.236	0.178	ns	−0.018	0.918	ns
BMR (kcal)	−0.210	0.232	ns	−0.195	0.269	ns	0.101	0.571	ns
PhA (°)	0.156	0.378	ns	0.042	0.814	ns	−0.646	<0.001*	*p* < 0.0036^**^

*p < 0.05; ns, not significant after Bonferroni correction; **significant after Bonferroni correction.

Additional examination of the correlation between ΔECW/ICW, ΔPhA, and AMS symptom scores showed that ΔECW/ICW had a negative connection with headache scores (*r* = −0.463, *p* = 0.006), but showed no relation to other symptoms. ΔPhA displayed a negative relationship with several scores: headache (*r* = −0.437, *p* = 0.001), gastrointestinal symptoms (*r* = −0.427, *p* = 0.012), fatigue/weakness (*r* = −0.392, *p* = 0.022), and dizziness/lightheadedness (*r* = −0.467, *p* = 0.005). After using Bonferroni correction, the correlation between ΔPhA and fatigue/weakness was not statistically valid anymore (Table 5). This indicates that the ΔPhA is inversely related to many main symptoms of AMS, while the ΔECW/ICW relates only to the headache score. This finding backs the idea that the level of cell function damage shown by ΔPhA might connect to the wide-ranging causes of AMS symptoms, whereas ΔECW/ICW might have a more precise link to changes in brain pressure or how high-altitude headaches work.

**Table 5:** Spearman correlation analysis between ΔECW/ICW, ΔPhA, and AMS-related symptom scores.

Symptoms	ΔPhA			ΔECW/ICW		
	*r*	*p*	Bonferroni correction (adjusted *α* = 0.0125)	*r*	*p*	Bonferroni correction (adjusted *α* = 0.0125)
Headache	−0.437	0.010*	*p* < 0.0125^**^	−0.463	0.006*	*p* < 0.0125^**^
Gastrointestinal symptoms	−0.427	0.012*	*p* < 0.0125^**^	−0.162	0.361	ns
Fatigue and/or weakness	−0.392	0.022*	ns	−0.286	0.101	ns
Dizziness/lightheadedness	−0.467	0.005*	*p* < 0.0125^**^	−0.18	0.309	ns

*p < 0.05; **significant after Bonferroni correction; ns, not significant after Bonferroni correction.

### 3.6 Multiple linear regression analysis of factors influencing the LLSS score

An exploratory multiple linear regression analysis was conducted to examine the predictive significance of body composition parameters for AMS. Given the relatively small sample size (*n* = 34) and to ensure statistical power and model stability, adhering to the principle of including at least 10 samples per variable, a total of three variables were incorporated into the regression model. The predictors included ΔECW/ICW and ΔPhA, both of which were identified with *p* < 0.05 in the Spearman correlation analysis. Additionally, gender was adjusted for as a key confounding factor, considering its known clinical relevance to potential influences on LLSS scores. The LLSS score was used as the dependent variable. A multiple linear regression analysis was done to explore how body composition factors predict AMS. With only 34 samples, three variables were added to keep statistical power and model stable, following the rule of 10 samples per variable. The predictors were ΔECW/ICW and ΔPhA, both showing *p* < 0.05 in prior Spearman analysis. Gender was adjusted for as a key confounding factor, considering its known clinical relevance to potential influences on LLSS scores. The LLSS score served as the dependent variable. Model diagnostics revealed that the standardized residuals were normally distributed (*p* > 0.05) and homoscedastic. The overall model was significant (*R*^2^ = 0.440, Adjusted *R*^2^ = 0.384, F(3, 30) = 7.849, *p* = 0.001), with no multicollinearity (VIF < 2.0). Among the predictors, ΔPhA was an independent and significant negative predictor of the LLSS score (*β* = −0.507, *t* = −3.521, *p* = 0.001). Gender and ΔECW/ICW were not significant predictors (Table 6).

**Table 6:** Multiple linear regression analysis of factors influencing the LLSS score (*n* = 34).

Predictor	*B*	SE	*β*	*t*	*p*	VIF
(Constant)	2.684	0.454		5.91	0.000	
Gender	−0.593	0.506	−0.162	−1.173	0.250	1.023
ΔECW/ICW	−36.266	19.037	−0.276	−1.905	0.066	1.125
ΔPhA	−6.228	1.769	−0.507	−3.521	0.001^*^	1.109

*R*^2^ = 0.440, Adjusted *R*^2^ = 0.384, F(3, 30) = 7.849, *p* = 0.001; *p < 0.05.

## 4 Discussion

The incidence of AMS following rapid ascent to high altitude varies depending on altitude, rate of ascent, and individual factors, with reported rates ranging from 30 to 80% (6, 24, 25). In our study, which observed 34 volunteers exposed to 3,680 m for 24 h, the AMS incidence was 61.76%. In the uncorrected analysis, we found that in the initial phase, both ECW/ICW and PhA decreased compared to the plain-level values, while FFM increased. No significant changes were observed in body weight, BMI, ECW, ICW, TBW, FAT, protein, mineral, SMM, PBF, and BMR. These results suggest that during the initial phase of acute high-altitude exposure, before significant change in TBW, internal water redistribution and cellular functional impairment are already present. This process precedes and is independent of classic systemic water and sodium retention. One study found no significant changes in total body water on day 4 or day 8 of acute high-altitude exposure, which suggests possible water redistribution to interstitial or intracellular spaces (26), similar to our findings. However, Santangelo et al. (21) observed no difference in ECW/ICW in 15 healthy mountaineers 24 h after a 4-day trek from 1,164 m to 4,556 m. This discrepancy with our finding of decreased ECW/ICW might be explained by differences in the mode and rate of ascent. Regli IB found PhA decrease after 6 h in high altitude (14). Our PhA measure at 24 h still shows a decrease from sea level values, suggesting PhA decrease maybe begins within hours after rapid ascent to high altitude. FFM rose early in high altitude without weight and TBW change, maybe because BIA measures FFM wrong. Additionally, the observed increase in FFM in the early phase of high-altitude exposure, in the absence of significant changes in total body weight and TBW, might be attributed to estimation bias in BIA-derived FFM. BIA algorithms assume constant body water distribution, and this assumption may be violated during water shifts (27). Therefore, the increase in FFM should be interpreted as an indirect signal of intracellular edema rather than a true increase in lean tissue, which is inherently consistent with the finding of a decreased trend in ECW/ICW. But after Bonferroni correction analysis, we did not observe a statistically significant difference in ECW/ICW. This means that the observed effect may be a chance finding or that its effect size may be small, with insufficient stability in the current sample size of this study. Similarly, FFM also lacked statistical significance after correction, indicating that changes in muscle mass may be subtle or require a larger sample size for confirmation. In summary, the significant decrease in PhA remained robust even after stringent correction. We believe that impaired cellular functional integrity may represent the most prominent and consistent physiological response during the early stage of acute high-altitude exposure.

Intergroup comparison of static body composition only revealed that the AMS + group exhibited a lower trend in ECW/ICW (*t* = −2.14, *p* = 0.040) after acute high-altitude exposure. No significant differences were found in other body composition parameters, whether measured on the plain or on the high altitude. The negative results of BW, FFM, TBW, ICW and ECW in our study were similar to the finding of Zhou et al. (28). After using Bonferroni correction, the trend value of ECW/ICW was not shown. And this trend may need to be further explored in future studies with larger samples. We consider that relying solely on static body composition parameters measured at different time points may not reliably differentiate AMS status. Further comparison of the Δ value showed that the AMS + group had significantly greater decreases in both ECW/ICW and PhA. After Bonferroni correction, the difference in ΔECW/ICW was no longer significant, while the difference in ΔPhA remained obvious. Spearman correlation analysis revealed a negative correlation between ΔECW/ICW (*r* = −0.447, *p* = 0.008) and ΔPhA (*r* = −0.646, *p* < 0.001) with the LLSS score. However, the correlation value of ΔECW/ICW was not displayed even after applying Bonferroni correction. These findings suggest that the degree of water shift between inside and outside cells may not be the most specific characteristic of AMS, or its effect may be relatively weak. The early mechanism of AMS may lean more toward the degree of cellular-level hypoxic injury.

In a multiple regression analysis, after correcting for gender (29), only ΔPhA was found to predict the LLSS score independently (*β* = −0.507, *t* = −3.521, *p* = 0.001), ΔECW/ICW was not significant in this model. With gender and ΔECW/ICW accounted for, a decrease of one standard deviation in ΔPhA led to a 0.507 standard deviation increase in the LLSS score. ΔPhA directly quantifies the extent of cellular damage caused by hypoxia and encapsulates more upstream pathological information than ΔECW/ICW, thereby exhibiting stronger predictive power. We speculate that this impairment in cellular function may be attributable to acute hypoxia, which could directly inhibit mitochondrial ATP synthesis in the early stages. This inhibition may lead to the failure of the Na^+^/K^+^-ATPase (sodium–potassium pump) on the cell membrane, impairing its ability to maintain normal ionic gradients. Consequently, changes in cell membrane permeability and intracellular osmotic pressure may occur, ultimately resulting in cellular dysfunction (30–32). Our findings indicate that cellular functional integrity is a more central factor than water distribution in predicting AMS severity. This discovery advances the pathophysiological understanding of AMS from water distribution imbalance to a new level involving direct cellular injury. It highlights the potential of ΔPhA as a non-invasive, quantitative biomarker for predicting high-altitude reactions. Monitoring ΔPhA could enable the early and sensitive identification of high-risk individuals who react severely to hypoxic stress and have poor resistance, thereby facilitating early warning and targeted protection against AMS. Furthermore, monitoring ΔPhA could serve as a novel endpoint for evaluating the efficacy of interventions (17).

Although this study was exploratory in nature, the Bonferroni correction was intentionally adopted to provide more focused and mature insights by narrowing the analysis to variables that truly warrant greater attention. By setting a corrected *α*, we prioritized the control of Type I errors, thereby increasing confidence that susceptible individuals with AMS showed a significantly greater decrease in PhA. We acknowledge that the Bonferroni method is conservative and can increase the risk of Type II errors (false negatives), potentially masking effects of smaller magnitude. This may explain why the difference in ECW/ICW did not retain statistical significance after correction. This outcome underscores the exploratory nature of our analysis for variables other than ΔPhA and suggests that observed trends (e.g., in ΔECW/ICW) warrant validation in larger cohorts. In our study, Bonferroni correction is methodologically justified because the need for multiplicity adjustment arises from the number of statistical hypotheses tested concurrently on a family of related dependent variables, not merely from the number of independent groups being compared (33, 34). Our decision aligns with previous study where Bonferroni correction was applied to multiple outcome measures despite involving only two comparison groups to ensure stringent inference (35). Ultimately, this conservative statistical stance reinforces the reliability of our primary conclusion regarding ΔPhA as the most salient early marker of cellular response to acute high-altitude exposure.

This study had several limitations. First, conducting research in high-altitude environments presents numerous challenges. These included difficulties in maintaining laboratory-grade measurement precision and standardizing confounding factors (e.g., diet, physical activity, water intake), which reduces experimental control. Second, the sample size was relatively small. This likely resulted in low statistical power to detect small-to-moderate effects for non-significant results. The findings without significant differences should be interpreted with caution. Although the multiple linear regression analysis was limited to three core variables and yielded relatively robust results through rigorous statistical strategies, our findings should be considered preliminary and exploratory, requiring validation in larger cohorts. Third, we need to recognize the limitations of BIA technology. The measurement results obtained from this technique are indirect estimates based on the electrical properties of the human body, and its accuracy may be affected by the rapid physiological changes involved in this study. Finally, this study primarily focused on changes in body composition at 24 h after arrival at high altitude and did not capture the evolutionary trajectory over a longer time window. Future research could incorporate repeated assessments at multiple longitudinal time points (e.g., 6, 12, 24, 48, and 72 h) to comprehensively delineate the full relationship between the trajectory of body composition changes and the dynamic evolution of AMS symptoms, and to validate the predictive ability of ΔPhA and other indicators for delayed-onset or persistent AMS. Additionally, combining serum biomarkers (such as HIF-1α, IL-6, TNF-α, IL-1β, etc.) (36–39) could further clarify the direct link between ΔPhA, cellular edema, and AMS.

## 5 Conclusion

After rapid exposure to high altitude, body water often shifted into cells and pronounced cellular-level dysfunction occurred. Susceptible individuals with AMS exhibited a more pronounced decrease in PhA. The change of PhA (ΔPhA), rather than the change of ECW/ICW (ΔECW/ICW), was an independent predictor of AMS severity. Direct hypoxic injury to the cells may play a central role in the development of AMS. Monitoring ΔPhA may provide an effective, non-invasive method for early risk warning of AMS.

## Data Availability

The original data presented in the study are included in the supplementary materials (Supplementary Tables 1, 2), further inquiries can be directed to the corresponding author.
